# Enzymatic Catalysis in Favor of Blocky Structure and Higher Crystallinity of Poly(Butylene Succinate)-Co-(Dilinoleic Succinate) (PBS-DLS) Copolymers of Variable Segmental Composition

**DOI:** 10.3390/ma15031132

**Published:** 2022-02-01

**Authors:** Martyna Sokołowska, Jagoda Nowak-Grzebyta, Ewa Stachowska, Miroslawa El Fray

**Affiliations:** 1 Department of Polymer and Biomaterials Science, Faculty of Chemical Technology and Engineering, West Pomeranian University of Technology, Al. Piastow 45, 71-311 Szczecin, Poland; mm31747@zut.edu.pl; 2 Institute of Materials Technology, Poznan University of Technology, ul. Piotrowo 3, 60-965 Poznan, Poland; jagoda.pa.nowak@doctorate.put.poznan.pl; 3 Division of Metrology and Measurement Systems, Institute of Mechanical Technology, Poznan University of Technology, ul. Piotrowo 3, 60-965 Poznan, Poland; ewa.stachowska@put.poznan.pl

**Keywords:** poly(butylene succinate), PBS copolymer, enzymatic synthesis, CALB, block copolymers

## Abstract

To systematically investigate the synthesis of poly(butylene succinate)-co-(dilinoleic succinate) (PBS-DLS) copolymers and to enrich the library of polyesters synthesized via a sustainable route, we conducted a two-step polycondensation using fully biobased monomers such as diethyl succinate (DS), 1,4-butanediol (1,4-BD) and dilinoleic diol (DLD) in diphenyl ether, using *Candida Antarctica* lipase B (CAL-B) as biocatalyst. A series of PBS-DLS copolyesters with a 90-10, 70-30 and 50-50 wt% of hard (PBS) to soft (DLS) segments ratio were compared to their counterparts, which were synthesized using heterogenous titanium dioxide/silicon dioxide (TiO_2_/SiO_2_) catalyst. Chemical structure and molecular characteristics of resulting copolymers were assessed using nuclear magnetic spectroscopy (^1^H- and ^13^C-NMR) and gel permeation chromatography (GPC), whereas thermal and thermomechanical properties as well as crystallization behavior were investigated by differential scanning microscopy (DSC), dynamic mechanical thermal analysis (DMTA), digital holographic microscopy (DHM) and X-ray diffraction (XRD). The obtained results showed that, depending on the type of catalyst, we can control parameters related to blockiness and crystallinity of copolymers. Materials synthesized using CAL-B catalysts possess more blocky segmental distribution and higher crystallinity in contrast to materials synthesized using heterogenous catalysts, as revealed by DSC, XRD and DHM measurements.

## 1. Introduction

In recent decades, worldwide polymer production and consumption has relied on petroleum-based materials, whose processing exerts a negative impact on the environment and ecosystems, including CO_2_ emission, depletion of fossil resources and accumulation of waste. Since these undesirable effects led to critical values at the end of the 20th century, the development of biodegradable materials and sustainable chemistry has become the main priority in academia and industry [1]. In these circumstances, biomass feedstocks seem to have enormous potential as an inexhaustible source of carbohydrates, whose processing into biobased monomers or polymers can bring more positive aspects, such as lower energy consumption and greenhouse gas emissions. Among those biobased materials that may be taken under consideration, aliphatic polyesters exhibit several desirable properties, and they can be produced from plant-derived monomers, which is associated with a low environmental impact upon disposal. Due to their high mechanical strength, barrier properties, good processability and high capacity for polymer chain orientation, they have found potential applications in many areas. Moreover, the physicochemical properties of polyesters can be easily modified by using adequate additives or adapting processing methods to meet the appropriate material requirements. For example, improved gas barrier properties of fully biobased poly(ethylene 2,5-furandicarboxylate) (PEF) made it suitable for use as bottles and foils in the food and beverage industry [2]. On the other hand, polylactide (PLA) has found application in the packing industry because of the improvement in physical stability and processability [3]. Another valuable approach to improve the processability and functionality of biobased polymers is the formation of segmented block copolyesters, that consist of different types of segments. In this kind of materials, we can distinguish soft and hard phases, which possess different distinct transition temperatures. Soft segments impart elastomeric properties due to the low elasticity modulus and glass transition temperature (Tg), which are responsible for material flexibility. On the other hand, hard segments are responsible for dimensional and mechanical stability [4].

Growing interest in the conversion and utilization of biomass and rapid development in the biorefinery industry bring a wide spectrum of new opportunities in the production of novel monomers and polymers from renewable sources. Poly(butylene succinate) (PBS) is a semicrystalline aliphatic polyester synthesized by direct polycondensation of succinic acid (SA, or its diester) and butanediol (BD). In recent years, these monomers were entirely derived from crude oil resources, but the increasing demand to reduce environmental pollution led to the discovery that they can also be obtained from biomass feedstocks [5]. Considering these facts, we can assume that problems connected with monomers can be solved, but questions remain regarding the catalyst. Commonly, PBS materials are produced using antimony-based catalysts in the form of oxide or acetate, which present high performance and can work in elevated temperatures; however, residual metals remaining in the polymeric material are difficult to remove due to the strong metal-ester interactions and can cause material discoloration. This is a major disadvantage, as those metals can contribute to undesirable environmental impacts upon material disposal [6,7]. Moreover, this occurrence may disqualify polymer use in several applications, such as electronics and the medical industry. To circumvent this problem, new catalyst systems are needed to improve material applicability without inducing harmful environmental effects. One promising substitute for antimony compounds is a new generation of titanium-based catalysts, titanium dioxide/silicon dioxide (TiO_2_/SiO_2_), whose activity was found to be even higher with respect to antimony catalysts [8]. The heterogenous nature of this catalyst enables its removal from the polymer after synthesis, which precludes metal-ester interaction and material discoloration.

Enzymatic polymerization seems to be an interesting and alternative approach regarding polymeric materials and has a chance to replace conventional techniques and physical modification methods [9]. It exhibits a number of advantages, including high efficiency, substrate specificity, catalyst recyclability, and the ability to work in organic solvents and under mild reaction conditions [10]. Enzymes are classified into six groups: isomerases, ligases, oxidoreductases, transferases, lyases and hydrolases. Among these, hydrolases are most widely used in practice, mainly due to their stability during isolation and separation steps from living organisms. Recently, many types of polymers have been successfully synthesized via enzymatic polymerization, including polyesters such as succinate-based polyesters [11,12], 2,5-bis(hydroxymethyl)furan-based polyesters [13,14], vegetal oil-based polyesters [15], sugar-based polyesters [16] and 3,6-dianhydrohexitol-based polyesters [17].

*Candida antarctica* lipase B (CAL-B) is the most favorable enzymatic biocatalyst. The immobilized form on acrylic resin exhibits broad substrate specificity, high thermal stability up to 150 °C and catalytic reactivity. The increasing interest in CAL-B is also due to the extremely high enantio-, regio- and stereoselectivity, which allows for optically pure materials with well-defined structure [6]. In our previous paper, we confirmed this effect by comparing poly(butylene-succinate)-co-(dilinoleic succinate) (PBS-DLS) with a 7030 wt% hard to soft segment ratio, synthesized using CAL-B and a heterogenous TiO_2_/SiO_2_ catalyst [18]. The obtained results showed that by using CAL-B, the obtained copolymer was characterized by a more blocky structure, an altered microstructure, and higher crystallinity. Therefore, considering these interesting results, we decided to investigate the influence of the catalyst system on PBS-DLS copolymers possessing both higher and lower DLS soft segment content.

In this study, in order to build upon our previous work, we synthesized PBS-DLS copolyesters with 90-10 and 50-50 wt% hard to soft segment ratios, using both immobilized CAL-B and titanium dioxide/silicone dioxide (TiO_2_/SiO_2_) catalysts. The obtained materials were characterized using ^1^H-, ^13^C NMR, ATR-FTIR, GPC, DSC, DMTA, XRD and a digital holographic microscope. This way, when combined with the results from [18], we obtained a comprehensive view of the interaction between the effect of catalyst type and segmental composition on the physico-chemical properties of the copolymers.

## 2. Materials and Methods

The following chemicals were acquired from Sigma-Aldrich (Poznan, Poland): dichloromethane (DCM; ≥99.5%) and diphenyl ether (DE; ≥99%). Diethyl succinate (DS; ≥99%) was ordered from Matrix Chemicals (Sevelen, Switzerland). Succinic acid (SA; ≥99%) and 1,4-butanediol (BD; ≥99%) were acquired from Alfa Aesar (Kandel, Germany). Dimer linoleic diol (DLD; ≥96.5%) (trade name: Pripol™ 2033) was obtained from Croda Coatings & Polymers (Gouda, The Netherlands). Chloroform (≥98.5%) was acquired from Chempur (Piekary Slaskie, Poland), and methanol (≥99.8%) was bought from Stanlab (Lublin, Poland). The titanium dioxide/silicon dioxide catalyst (TiO_2_/SiO_2_) was bought from Huntsman (Germany). *Candida Antarctica* lipase B (CAL-B) covalently immobilized on polyacrylate beads (300–500 µm; ≥95%, Fermase CALB™ 10,000), with a nominal activity of 10,000 PLU/g (propyl laurate units per gram dry weight), obtained from Fermenta Biotech Ltd., Mumbai and Enzyme Catalyzed Polymers LLC (Akron, OH, USA). CALB was pre-dried under vacuum for 24 h at 40 °C, and diphenyl ether was stored over 4Å molecular sieves before use. The rest of the chemicals were used as received.

Poly[(butylene succinate)-co-(dilinoleic succinate)] (PBS-DLS) copolyesters with a 90-10 and 50-50 wt% hard to soft segments ratio were synthesized via two different procedures and catalytic systems. In the first procedure, a two-step enzymatic polycondensation in diphenyl ether was conducted, based on a previously described protocol [18]. Copolymers obtained by this method were abbreviated as PBS-DLS 90-10_E and PBS-DLS 50-50_E. In the second procedure, a two-step polycondensation in the melt was performed following a previously described method using a C-94 catalyst [18,19]. Herein, copolymers synthesized by this procedure were abbreviated as PBS-DLS 90-10_M and PBS-DLS 50-50_M. Additionally, for comparison, we include data for PBS-DLS 70-30 copolymers synthesized using CAL-B and TiO_2_/SiO_2_ catalysts in our previous work [18] and abbreviated them as PBS-DLS 70-30_E and PBS-DLS 70-30_M, respectively.

^1^H- and ^13^C-NMR spectra were recorded on a Bruker DPX 400 spectrometer (400 MHz, 1 s relaxation delay, 128 scans for ^1^H-NMR and 5120 scans for ^13^C-NMR) (Bruker Corporation, Massachusetts, USA). The samples were dissolved in CDCl_3_, and reported chemical shifts were referenced to the resonances of tetramethylsilane (TMS).

Attenuated Total Reflection Fourier Transform Infrared (ATR-FTIR) spectra were recorded on a Bruker ALPHA spectrometer (Bruker Corporation, Massachusetts, USA) across a spectral range of 400–4000 cm^−1^ and resolution of 2 cm^−1^. A total of 32 scans were performed for each sample; samples were vacuum dried before measurement.

The number-average (M_n_) and weight-average (M_w_) molecular weights were determined by gel permeation chromatography (GPC). Measurements were performed on the Agilent 1200 modular HPLC series system with a refractive index detector. The system was equipped with two PLgel 5 μm MIXED-C columns (300 × 7.5 mm). Calibration was performed on 12 polystyrene standards with masses (Mp) in the range 474–1,800,000 g/mol. Measurements were made at 35 °C. Chloroform (CHCl_3_) HPLC grade with a flow rate of 0.7 mL/min was used as the mobile phase. The samples with a concentration of 3 mg/mL were filtered through a PTFE membrane with a pore size of 0.2 µm before analysis. Data was recorded using the “ChemStation for LC” program and analyzed using the “ChemStation GPC” Data Analysis Software (Agilent, Santa Clara, USA).

The thermal transitions of obtained materials were evaluated using a TA Instruments DSC Q2500 Discovery differential scanning calorimeter (DSC)(TA Instruments Inc., New Castle, DE, USA). The heating-cooling rates were 10 °C/min, and the measurements were carried out over the temperature range of –90 to 200 °C under N_2_ atmosphere. The glass transition temperature (T_g_) was calculated as a midpoint of the transition during a second heating step.

The X-ray diffraction (XRD) patterns of PBS-DLS polymeric films were recorded by Empyrean PANalytical X-ray diffractometer (Malvern Panalytical, Malvern, UK), using Cu K (λ = 0.154 nm) as the radiation source in the 2*θ* range 10–60, with a step size of 0.026. The crystallinity index (X_c_’) was calculated from the XRD profiles from the ratio between the crystalline peaks area (A_c_) and area of all peaks (crystalline and amorphous) (A_t_).

The microstructure of materials during spontaneous crystallization was assessed using a Lyncée Tec DHM T1000 digital holographic transmission microscope (Lyncée Tec., Lausanne, Switzerland). Holographic off-axis geometry was realized by a Mach-Zehnder interferometer and 666 nm laser diode emitting linear polarized beam with a small illumination power of 200 µm/cm^−2^. The holograms were recorded using a CCD camera (1024 × 1024 pixels, 30 fps). Two magnifications were used: 10× (objective NA = 0.3; FOV = max 660 µm; no immersion) and 50× (objective NA = 0.75; FOV = max 180 µm; no immersion). The lateral resolution was 0.1 µm, and the axial resolution was below 1 nm. Data were collected and evaluated using the Koala software of Lyncée Tec. Before measurement, the samples were placed between two microscope slides, heated to 200 °C, cooled to room temperature and again reheated to 200 °C to start measurement, in which crystallization behavior was observed during cooling to room temperature. The thickness of the samples between the slides was 6–15 µm.

To determine the torsion modulus and the glass transition temperatures, samples for dynamic mechanical and thermal analysis (DMTA) were prepared by melt-pressing at a temperature range from 90 to 115 °C. The specimens were 100 µm thick, 1 mm wide and 50 mm long. Measurements were performed in tensile mode using a DMA Q800 apparatus from TA instruments (TA Instruments Inc., New Castle, DE, USA). Analysis was conducted at a constant frequency of 1 Hz, heating rate of 2 °C/min and amplitude of 60.

## 3. Results

**Figure 1:**
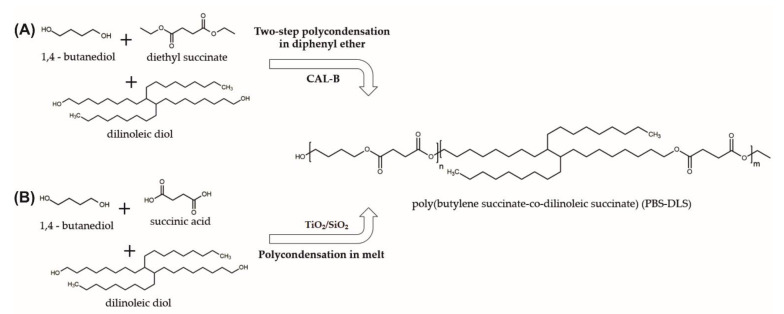
Synthesis of poly(butylene succinate-co-dilinoleic succinate) (PBS-DLS) copolymer via (**A**) two-step polycondensation in diphenyl ether using CAL-B and via (**B**) polycondensation in melt using TiO_2_/SiO_2_ catalyst. (n—90, 70 or 50 wt% of hard segments; m—10, 30 or 50 wt% of soft segments).

In the present work, fully biobased PBS-DLS copolymers containing poly(butylene succinate) (PBS) as the hard segments and poly(dilinoleic succinate) (DLS) as the soft segments were successfully synthesized via two-step polycondensation in diphenyl ether using CAL-B as a biocatalyst and via the polycondensation method in melt using a TiO_2_/SiO_2_ catalyst (Figure 1). To investigate the influence of the DLS soft segment content on the material properties, copolymers with different ratios of hard to soft segments (90-10, 70-30 and 50-50 wt%) were produced. After synthesis, all materials were recovered within high reaction yields (≥80%).

As shown in Figure 1, to produce PBS-DLS copolymers using CAL-B as a catalyst, we choose to perform polycondensation reaction in the solvent using diethyl succinate instead of succinic acid. There is no doubt that succinic acid is a popular and effective building block for polyester synthesis; however, it exhibits a high melting point (T_m_ = 184 °C) and relatively low solubility in 1,4-butanediol. Thus, during polycondensation reactions, high temperatures need to be applied (≥184 °C) [6]. Therefore, during enzymatic reactions, which are normally conducted in lower temperatures to avoid enzyme deactivation (herein 95 °C), problems such as phase separation and low molecular weight of synthesized products occur [20]. To circumvent phase separation during CAL-B-catalyzed polycondensation, diethyl succinate was used as an acyl donor. Moreover, according to the data presented in the literature, enzymatic polycondensation in diphenyl ether made it possible to obtain products with relatively higher molecular weight, longer chains, and desirable molar composition [6,21].

**Figure 2:**
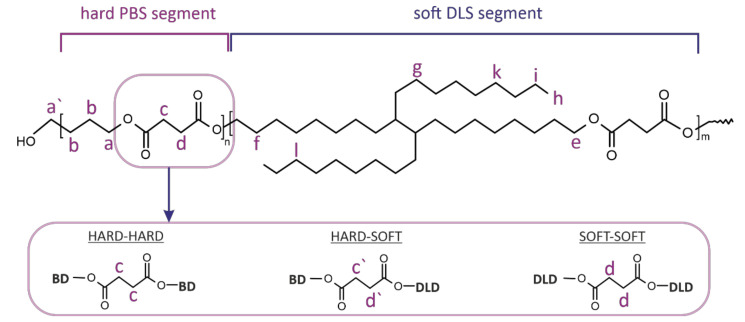
Chemical structure of poly[(butylene succinate)-co-(dilinoleic succinate)] copolyester, with the possible locations of hard and soft segments.

**Figure 3:**
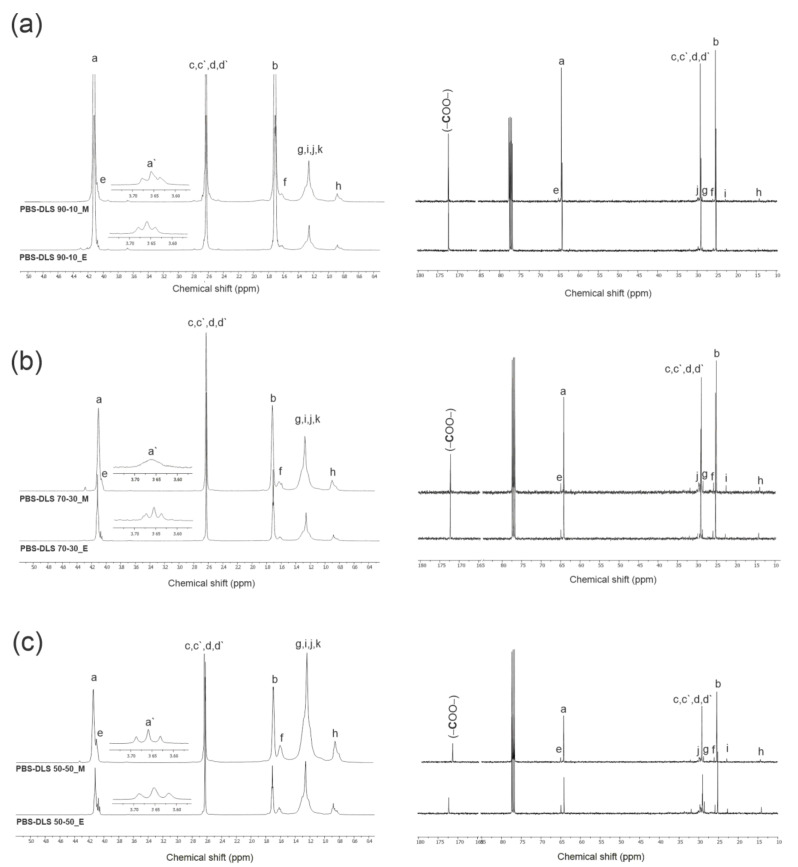
^1^H- and ^13^C NMR spectra of PBS-DLS copolymer series containing (**a**) 90 wt% (**b**) 70 wt% with permission from [18] Wiley, Copyright © 2020 Sokołowska M. et al. (**c**) 50 wt% of hard PBS segment.

To confirm the chemical structure of obtained copolymers, ^1^H- and ^13^C NMR spectra were recorded based on which detailed structural analysis of PBS-DLS series was conducted (Figure 2 and Figure 3).

Figure 3 depicts ^1^H- and ^13^C- NMR spectra of block PBS-DLS copolyesters containing 90, 70 and 50 wt% of hard PBS segments. The detailed NMR assignments are as follows: ^1^H NMR (400 MHz, CDCl_3_-*d_1_*, ppm): 4.12 (a) (4H, –O–CH**_2_**–, from 1,4-BDO), 4.06 (e) (4H, –O–CH_2_–, from DLD), 3.65 (a′) (–CH_2_–OH, end group from 1,4-BDO), 2.63 (c,c′,d,d′) (4H, –CO–CH_2_–, from DS/SA), 1.71 (4H, –O–CH_2_–CH_2_–, from 1,4-BDO), 1.60 (f) (4H, –O–CH2–CH_2_–, from DLD), 1.24 (g,i–k) (–CH_2_–, from DLD), 0.87 (h) (6H, –CH2–CH_3_, end group of DLD); ^13^C NMR (400 MHz, CDCl_3_-*d_1_*, ppm): 172.27 (–COO–), 64.17 (a) (–CO–O–CH_2_, from 1,4-BDO), 25.22 (b) (–CO–O–CH_2_–CH_2_–, from 1,4-BDO), 29.01 (c,c′,d,d′) (–CO–O-CH_2_–, from DS/SA) 64.95 (e) (–CO–O–CH_2_, from DLD), 25.90 (f) (–CO–O–CH_2_–CH_2_–, from DLD), 14.13 (h) (–CH_2_–CH_3_, end group of DLD), 29.09 (j), 28.59 (g), 22.69 (i) (–CH_2_–, from DLD). Based on ^1^H- and ^13^C NMR analysis, the expected chemical structure of PBS-DLS copolyesters was confirmed. Signals (a) and (e) indicated ester bond formation between BD-DS/SA and DLD-DS/SA, and signals (f–k), related to the protons of methylene groups in the DLD long aliphatic chain, proved that soft segments were well incorporated into the material structure. Notably, with the higher content of soft segments, the intensities of the (e–k) signals characteristic for DLD increased.

**Figure 4:**
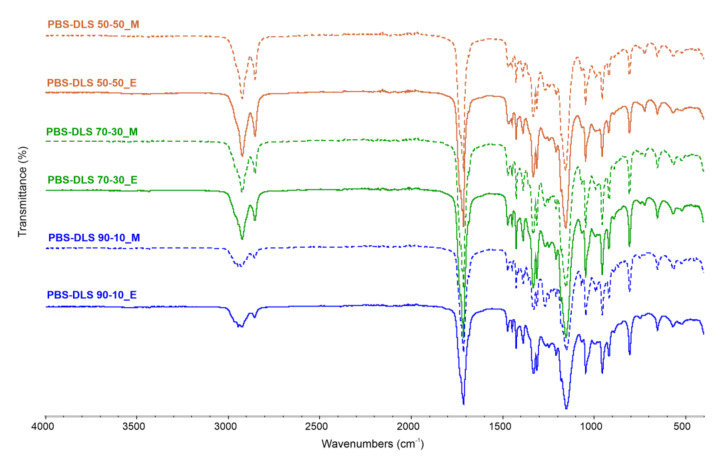
FTIR spectra of PBS-DLS copolyesters.

The expected PBS-DLS copolymer structure was also assessed by ATR-FTIR analysis and is presented in Figure 4. The characteristic absorption bands of the obtained materials are assigned as follows: ATR-FTIR (cm^−1^): 2925 and 2855 (C–H asymmetric and symmetric vibration in CH_2_), 1713 (C=O stretching vibrations), 1472, 1447, 1332–1426 (–CH– deformation and wagging vibrations), 1266 and 1153 (C–O–C asymmetric and symmetric stretching vibrations), 568–1046 (C–C and C–H in-plane and out-plane bending motions). Intensities of peaks at 2925 and 2855 cm^−1^, corresponding to the methylene groups, increased with the higher content of soft DLS segments, indicating their greater amount within the copolymer structure. This phenomenon was also confirmed by comparing the ratio of peak characteristics for –CH_2_– bonds to the C=O ester bonds derived from succinate, for which the molar fraction is the same in each copolymer. The calculated ratios reach greater values, with an increasing amount of soft segment content in both copolymer series. Additionally, the fingerprint region appearing at the spectral range from 568 to 1046 cm^−1^ did not indicate any differences between the two PBS-DLS copolymer series.

Additionally, the sensitivity of the ^13^C NMR enabled us to perform detailed structural analysis regarding segmental distribution within the copolyester microstructure. Due to the changes in the chemical environment of the methylene groups present between carbonyl carbon atoms in the DS sequence, it was possible to distinguish four possible monomer arrangements. For calculations, we used chemical shifts characteristic for protons of methylene groups between two hard segments (H−H, signal (c), 172.27 ppm), hard and soft segments (H−S/S−H, signals (c′,d′) 172.31 ppm) and two soft segments (S−S, signal (d), 172.35 ppm). Thus, the average sequence length (*L_S_, L_H_*) of soft and hard segments, respectively, as well as the degree of randomness *R* were calculated using Equations (1)–(3) [18,22,23].

(1) $$L_{H}=\frac{F_{H-H}+0.5\left(F_{H-S,\;\mathrm{tot}}\right)}{0.5\;(F_{H-S,\;\mathrm{tot}})}$$

(2) $$L_{S}=\frac{F_{S-S}+0.5\left(F_{H-S,\;\mathrm{tot}}\right)}{0.5\;(F_{H-S,\;\mathrm{tot}})}$$

(3) $$R=\frac{1}{L_{H}}+\frac{1}{L_{S}}$$

where F_x_ is the normalized integral value from ^13^C NMR (x = H−S,tot; H−H; S−S). The results calculated for each copolyester are presented in Table 1.

When the *R* value is 1, the distribution of the hard and soft segments within the copolymer structure is random. On the other hand, when the *R* value is lower than 1, the copolymer exhibits a block segment distribution [23].

**Figure 5:**
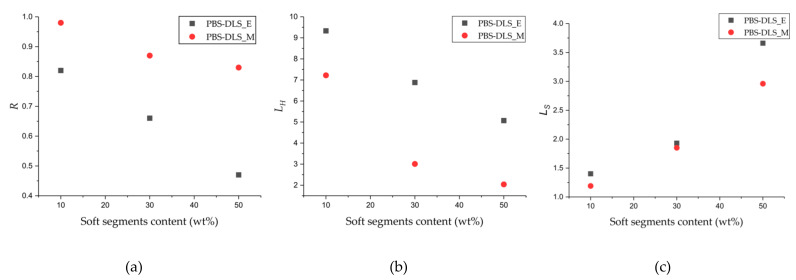
(**a**) Degree of randomness (R), average sequence length of (**b**) hard (L_H_) and (**c**) soft (L_S_) segments as a function of soft segment content in PBS-DLS_E and PBS-DLS_M copolyesters.

According to Figure 5 and the values presented in Table 1, the *R* values obtained for PBS-DLS copolyesters with 90-10, 70-30 and 50-50 wt% ratio of hard to soft segment synthesized using CAL-B reached values less than 1 in comparison to PBS-DLS_M copolyesters (0.82 vs. 0.98 in PBS-DLS 90-10; 0.66 vs. 0.87 in PBS-DLS 70-30; 0.47 vs. 0.83 in PBS-DLS 50-50), which indicates a more blocky segmental distribution within the PBS-DLS_E macromolecule structure. Moreover, when the *R* values for PBS-DLS_M materials change insignificantly, for the PBS-DLS_E they clearly decrease with an increasing content of soft segments, which indicates that the copolyester microstructure is becoming less and less random when we change the soft segment content from 10 to 30 and 50 wt%.

Furthermore, with increasing soft segment content, a decrease in *L_H_* and increase of the *L_S_* values appear, which is an additional confirmation that the long aliphatic sequences derived from DLD have been successfully incorporated into the copolyester microstructure. Interestingly enough, while the *L_S_* values are rather comparable for PBS-DLS_E and _M series, a greater discrepancy can be observed in the case of *L_H_* values. By comparing the two series of the obtained copolymers, we can observe that the average sequence length of hard segments, *L_H_*, is significantly higher for PBS-DLS_E copolymers; this may indicate that during polycondensation reaction, CAL-B is able to create hard units more effectively than soft units, and this phenomenon is probably related to the diol chain length. Enzymes have a catalytically active site pocket, the area of which is rather limited; thus, a diol with shorter chain length (herein, 1,4-BDO, number of carbon atoms = 4) can reach this region more easily in comparison to a diol with a relatively long aliphatic chain (herein DLD, number of carbon atoms = 36), which can hinder effective transesterification and its incorporation into the macromolecule structure at the early synthesis stages. Therefore, by using CAL-B, we can to some extent control the block structure, which is an indisputable advantage, as it directly affects the crystallinity of copolymer.

Moreover, based on the ^1^H NMR spectra, integration values of selected characteristic peaks arising from hard and soft sequences present in the copolyester structure were used to calculate the real content of each block and number average molecular weights (M_n_) using Equations (4)–(10):

(4) $$D=\frac{\frac{I_{1.71}}{n_{1.71}}}{\frac{I_{0.85}}{n_{0.85}}}$$

where D represents the ratio of PBS hard segments to DLS soft segments, I_1.71_ is the integral of the signal at 1.71 ppm, which is related to four protons characteristic for BD units (–CH_2_–) and I_0.85_ is the integral of the signal at 0.85 ppm, which arises from six protons in DLD units (–CH_3_). n_1.7_ and n_0.85_ represent a number of protons in BD and DLD units. The weight percentage of hard segments (%W_H_) of PBS-DLS copolyesters was computed from D using Equation (5):

(5) $${\%W}_{H}=\frac{D\cdot M_{h}}{D\cdot M_{h}+M_{s}}$$

where %W_H_ is the weight percentage of PBS hard segments, M_h_ is the molecular weight of hard segments (172 g/mol) and M_s_ is the molecular weight soft segments (624 g/mol). Likewise, using the signals of BD and DLD, we can compute the mole percent of each block using Equations (6) and (7):

(6) $$\left[{\%\mathrm{Mol}}_{h}\right]=\frac{\frac{I_{1.71}}{n_{1.71}}}{\frac{I_{1.71}}{n_{1.71}}+\frac{I_{0.85}}{n_{0.85}}}\cdot 100\%$$

(7) $$\left[{\%\mathrm{Mol}}_{s}\right]=\frac{\frac{I_{0.85}}{n_{0.85}}}{\frac{I_{1.71}}{n_{1.71}}+\frac{I_{0.85}}{n_{0.85}}}\cdot 100\%$$

where %Mol_h_ and %Mol_s_ are the mol percentages of hard and soft segments, respectively. 

Furthermore, by comparing the BD and DLD signals to that of the end groups, which are signals related to hydroxyl groups of BD at a chemical shift of 3.68 ppm (–CH_2_OH), it is possible to calculate the number of hard and soft segments (Num_h_ and Num_s_) using Equations (8) and (9). We can expect that the macromolecules are capped by a BD on either end, so there will be two end-groups (n_end_).

(8) $$\left[{\mathrm{Num}}_{h}\right]=\frac{\left(I_{1.71}\cdot n_{3.68}\cdot n_{\mathrm{end}}\right)}{I_{3.68}\cdot n_{1.71}}$$

(9) $$\left[{\mathrm{Num}}_{s}\right]=\frac{\left(I_{0.85}\cdot n_{3.68}\cdot n_{\mathrm{end}}\right)}{I_{3.68}\cdot n_{0.85}}$$

Finally, number average molecular weights of copolymers were calculated using Equation (10):

(10) $$\left[M_{n}\right]={\mathrm{Num}}_{h}\cdot M_{h}+{\mathrm{Num}}_{s}\cdot M_{s}$$

According to the values presented in Table 2, the weight and mole fractions of hard and soft segments were in good agreement with those estimated theoretically. Nevertheless, it should be noted that the PBS-DLS copolymers obtained with the use of CAL-B make it possible to obtain values that are similar to their initial feed ratios as compared to PBS-DLS copolymers synthesized using a heterogenous catalyst. In the PBS-DLS_M series, a greater amount of DLD soft units was incorporated into the copolymer structure in contrast to PBS-DLS_E copolymers. We speculated that this was due to the removal of 1,4-BDO from reaction media during polycondensation in the melt when a high vacuum was applied, as we discussed in our previous study [18].

Furthermore, based on ^1^H NMR and GPC, the variable amount of DLS soft segments and the synthesis method had a significant influence on the molecular weights of the aliphatic copolyesters. As shown in Table 2, polycondensation reaction performed using CAL-B catalyst yielded materials with relatively lower number (M_n_) and weight (M_w_) average molecular weights as compared to their counterparts synthesized using a heterogenous catalyst. In addition, it is clear from Table 2 that the molecular weights of the PBS-DLS copolyesters with 50 wt% DLS in the feed are the lowest. In enzymatic polycondensation, this may be due to the fact that CAL-B exhibits variable specificity for monomers differing in chain lengths, as previously reported by Jiang et al. [12]. They found that the M_n_ values of aliphatic polyesters increased with increasing chain length of the diacid ethyl esters from 2 to 4; however, further increasing of the chain length from 4 to 8, 10 or 12 yielded materials with lower molecular weights. In addition, Catalani et al. performed azeotropic polymerization using a CAL-B catalyst to obtain isosorbide-based polyesters [17]. Conducted studies revealed that M_w_ increased from 3000 to 21,000 g/mol when the chain length of diacid ethyl esters increased from 2 to 4, but those values reached 9000 g/mol when the chain length increased to 8. In our study, the chain length of dilinoleic diol (DLD) used for polycondensation is 36, which, considering the above deliberations may lead to lower molecular weight of the obtained materials due to the low specificity of the enzyme for the monomer possessing a relatively long aliphatic chain. Furthermore, if we look at polydispersity index (Đ) values, in CAL-B-catalyzed copolyesters, they vary from 3.1 to 8.2, depending on the content of DLS soft segments. From the point of the step-growth polycondensation process, these values are relatively high, and this may be due to the reason that CAL-B exhibits a higher tendency to produce copolyesters with a chemical structure that is more blocky (Table 1). On the other hand, PBS-DLS_M copolyesters reach the typical range, varying from 2.3 to 3.3, which is common for a step-growth polycondensation process with high conversion.

**Figure 6:**
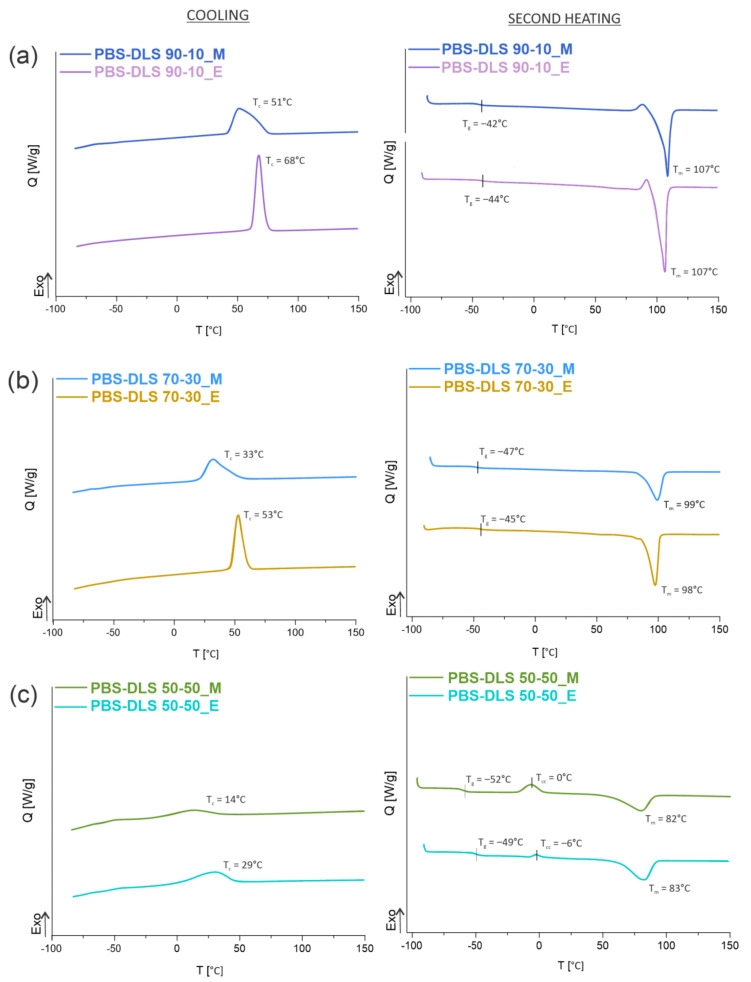
DSC cooling and second heating thermograms of PBS-DLS_E and PBS-DLS_M series with (**a**) 10 wt% (**b**) 30 wt% with permission from [18] Wiley, Copyright © 2020 Sokołowska M. et al. (**c**) 50 wt% content of soft segments.

**Figure 7:**
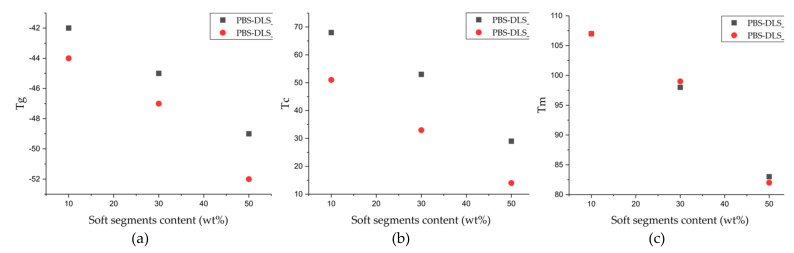
(**a**) Glass transition temperatures (T_g_), (**b**) crystallization temperatures (T_c_) and (**c**) melting temperatures (T_m_) of the obtained aliphatic PBS-DLS copolyesters as a function of soft DLS segment.

It is obvious that in block copolyesters, crystallization capacity of the hard segments favors the phase separation, while the soft segments are supposed to be amorphous to avoid crystallization. The combination of such two components leads to the formation of a thermally reversible multi-phase structure that is typical for thermoplastic elastomers (TPE). During crystallization, well-organized crystalline nanodomains embedded in the amorphous phase act as physical network nodes that impart dimensional, thermal and mechanical stability [24]. Herein, to investigate the effect of the soft segment content on PBS-DLS copolyesters, DSC analysis was conducted following the heat-cool-heat procedure and only cooling and second heating transitions were considered to provide comparable results. Moreover, the total crystalline phase content in the polymer (X_c_) was also evaluated using the following Equation (11):

(11) $$X_{c}=\frac{\Delta H_{m}}{\Delta H_{m}^{{}^{\circ}}}\cdot 100\%$$

where W_H_ is the weight amount of the hard segments, ΔH_m_ is the melting enthalpy of the PBS homopolymer or PBS-DLS copolymers, and ∆H_m_° is the melting enthalpy of 100% crystalline PBS (110.3 J/g) [25]. Obtained DSC values for copolyesters are collected in Table 3 and Figure 6 and Figure 7. 

According to the DSC data presented in Figure 6 and Figure 7 and Table 3, the microstructure of PBS-DLS copolymers consists of two phases: the crystalline phase, as evidenced by the visible melting point (T_m_), and the amorphous phase, formed by hard and soft segments as demonstrated by the glass transition temperature (T_g_). The presence of the T_m_ and T_g_ indicate a heterogenous microstructure of all PBS-DLS copolyesters, in which the chemical bonds between two segments prevent creating two neat phases. Generally speaking, herein we can distinguish one continuous amorphous phase in which crystalline domains are dispersed. However, this amorphous phase not only comprises flexible soft segments but also hard segments, which are not involved into crystalline structures, and their aliphatic chains are too short to demonstrate a clear glass transition temperature. In effect, on DSC thermograms, we observe only one clear T_g_, which reaches lower values with an increasing amount of DLS soft segments; this phenomenon is expected, as we introduce a greater amount of amorphous phase between PBS hard units. PBS-DLS copolyesters synthesized using CAL-B catalyst showed higher T_g_ values in comparison to PBS-DLS_M materials (−44 vs. −42 °C in PBS-DLS 90-10; −47 vs. −45 °C in PBS-DLS 70-30 and −52 vs. −49 °C in PBS-DLS 50-50), which is probably related to the fact that after polycondensation reaction performed with the use of TiO_2_/SiO_2_ catalyst we obtained copolymers with higher weight percentage of DLS soft units (Table 2). Moreover, melting temperature (T_m_), melting enthalpy (ΔH_m_), crystallization temperature (T_c_) and crystallization enthalpy (ΔH_c_) showed the following trend with respect to the soft segment content: T_m_, ΔH_m_, T_c_ and ΔH_c_ values decreased as the amount of DLS soft segments increased from 10 to 30 or 50 wt%, and we can observe this occurrence in both PBS-DLS copolymer series (Figure 7). Considering the T_m_ values obtained for PBS-DLS_E and PBS-DLS_M, we are not able to observe clear differences; however, significant discrepancies appear in the case of crystallization temperatures (T_c_), where greater values are obtained for PBS-DLS copolyesters synthesized using CAL-B (68 vs. 51 °C in PBS-DLS 90-10; 53 vs. 33 °C in PBS-DLS 70-30; 29 vs. 14 °C in PBS-DLS 50-50), which is also reflected by crystallization enthalpy. Interestingly enough, if we focus on the T_c_ exotherm regions corresponding to PBS-DLS_M copolymers, they are notably broader and flatter regarding the PBS-DLS_E materials, which leads to the conclusion that the crystalline phase in this case is less homogenous, and crystallites with variable size-distribution and degree of perfection are formed. In addition, we found that the total crystalline phase content (X_c_) decreased with an increasing amount of DLS soft segments in PBS-DLS copolyesters, and this can be explained by the decrease of chain regularity affected by a long and flexible DLD chain, which effectively hinders the formation of crystalline structures due to its amorphous nature. However, CAL-B-catalyzed materials reach higher X_c_ values in comparison to PBS-DLS copolyesters synthesized using a heterogenous catalyst.

To better understand the crystallization behavior of PBS-DLS copolyesters, digital holographic microscope (DHM) was used to record phase images. This method is not a common technique in polymer research; however, it has been applied to study copolymer brushes grafted on different surfaces [26] or modified poly(lactide-urethane) antifouling coatings [27]. However, our group for the first time described the use of a digital holographic microscope to study the optical properties of copolyesters [18].

**Figure 8:**
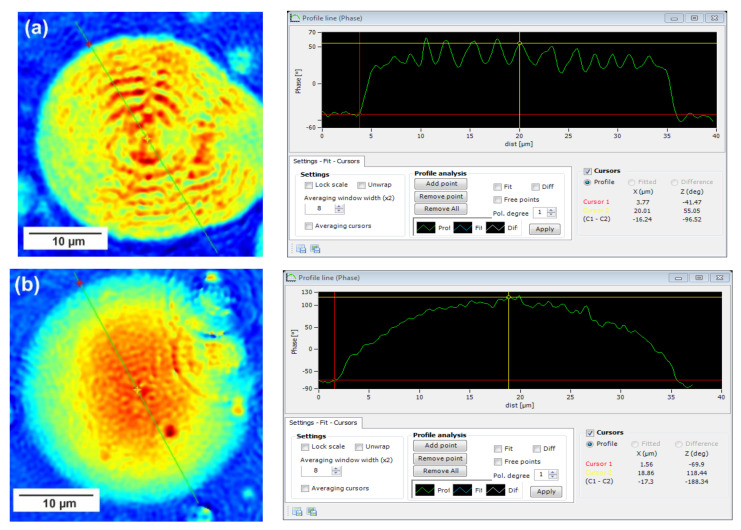
Holographic images and corresponding profiles of the phase changes along the green line recorded for a single spherulite crystalized from the melt for (**a**) PBS-DLS 90-10_E and (**b**) PBS-DLS 90-10_M. The DHM microscope magnification is 50×.F.

**Figure 9:**
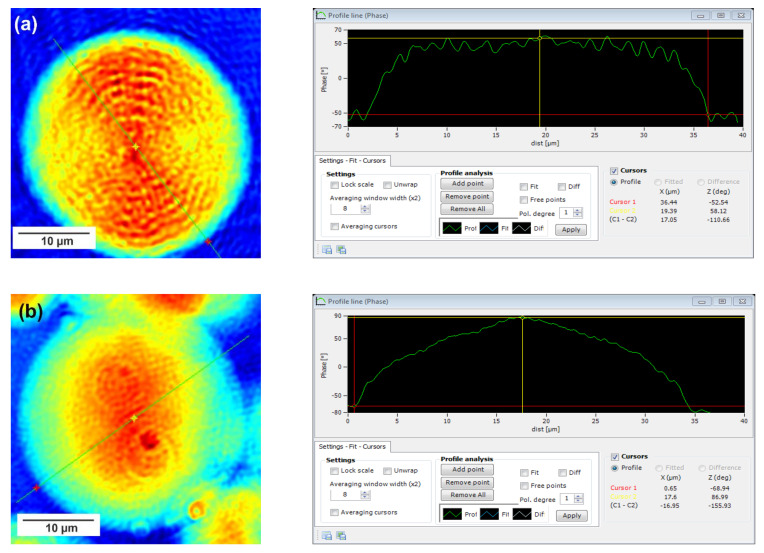
Holographic images and corresponding profiles of the phase changes along the green line recorded for single spherulites crystalized from the melt for (**a**) PBS-DLS 70-30_E and (**b**) PBS-DLS 70-30_M. The DHM microscope magnification is 50×.

**Figure 10:**
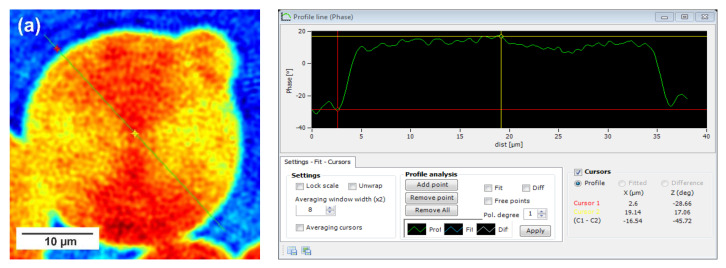

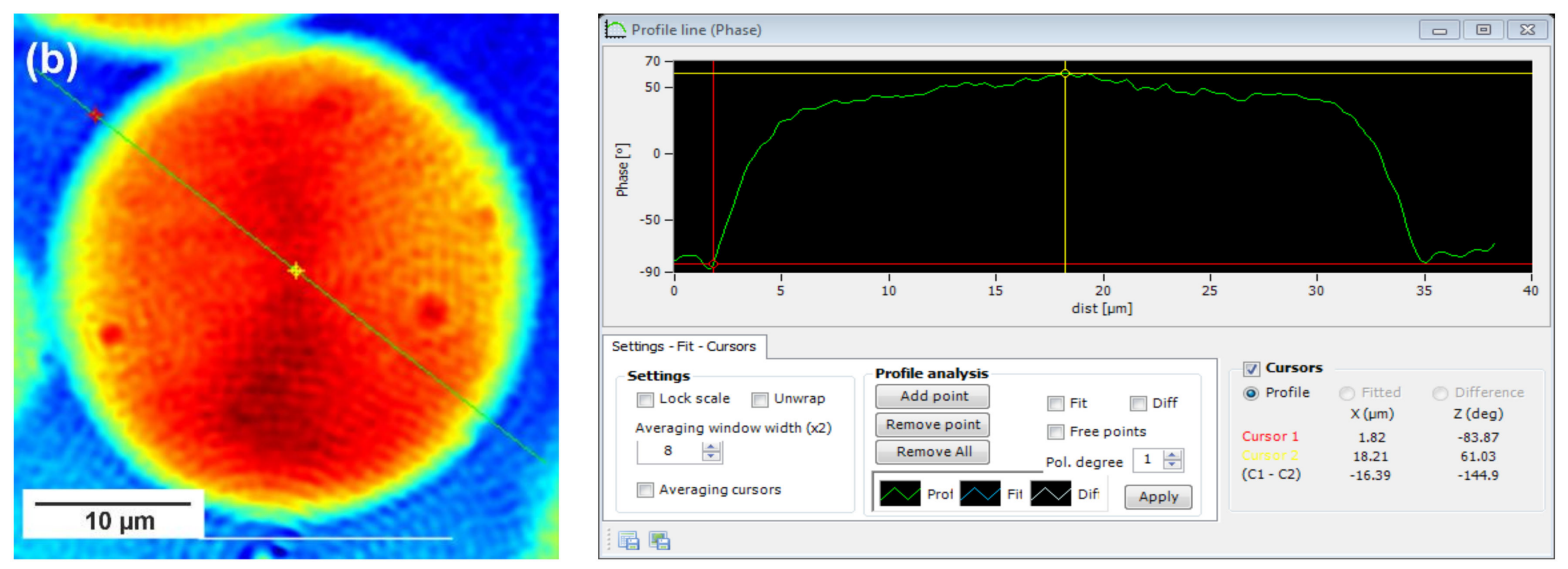
Holographic images and corresponding profiles of the phase changes along the green line recorded for single spherulites crystalized from the melt for (**a**) PBS-DLS 50-50_E and (**b**) PBS-DLS 50-50_M. The DHM microscope magnification is 50×.

In this technique, recorded profiles along the green lines visible on the phase images represent the one-dimensional distribution of optical way changes (Figure 8, Figure 9 and Figure 10). Optical way is the product of both refractive index and geometrical way. Refractive index depends on the density of the sample. Therefore, the observed phase change also gives information about the density distribution in the sample. Recorded profiles enabled us to measure differences in the phase change Δφ (in deg) when the laser beam passes through the crystallites, as compared to the phase when the beam is passing through the amorphous area. The scale marked on the *x*-axis is in µm.

The images presented in Figure 8, Figure 9 and Figure 10 show spherulite formation when the sample of PBS-DLS copolyester is spontaneously cooled to 24 °C. The figures show spherulites of similar diameter. Spherulites are the spherical semicrystalline structures that can be found during crystallization of polymers from the melt, and their morphology can be strongly affected by the number of nucleation agents, polymer structure and cooling rates [28]. Herein, the red color corresponds to the largest phase change of the beam passing through the sample, and the dark blue color to the smallest. Sample thickness was about 10 µm.

**Figure 11:**
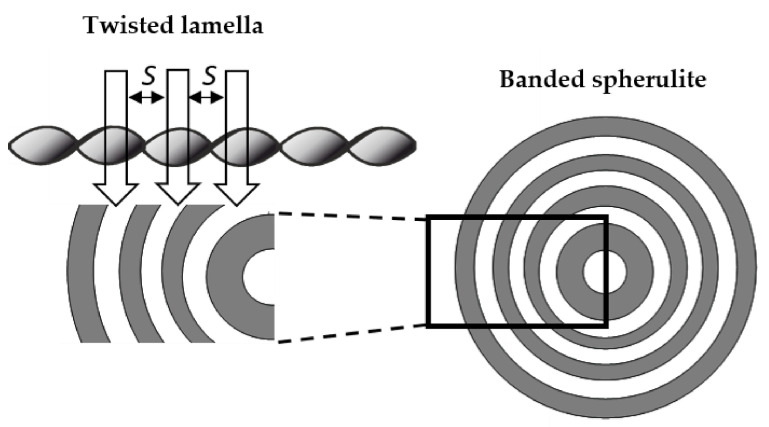
Scheme showing possible twisted helical chain orientation corresponding to the bright-dark rings in banded spherulites. S—band spacing.

Focusing on the morphology of the developed crystal structures, we can observe that CAL-B-catalyzed copolyesters exhibit the characteristic lamellar crystals radiating outwards from the center, as in all spherulites, and demonstrate alternative bright and dark bands (banded spherulites). This phenomenon is also clearly reflected by recorded profiles along the green lines, where regular band spacing (S) in PBS-DLS_E is detected. As the amount of soft segments increases, those structures are less pronounced, since the crystalline phase is not dominant. Herein, the banded structure most probably results from different birefringence connected with the twisting growth of lamellar crystals (see Figure 11), which can be affected by many factors. Experimental findings of Xu J. and colleagues revealed that the driving force for the twisting of polymer crystals is the anisotropic and unbalanced surface stresses, which are responsible for the handedness of lamellar twisting [29]. Nevertheless, there are still some challenges and uncertainties remaining in the area of polymer-banded spherulites that need to be explored to fully understand this phenomenon. However, there is no doubt that it occurs in the case of well-organized crystalline structures, where the bright birefringent regions can be attributed to the edge-on projection of crystalline lamella, while the dark regions correspond to the flat-on view. Interestingly enough, observed structures are not visible in the case of copolyesters synthesized using a heterogenous catalyst, which emphasizes that the type of catalytic system used during reaction has a significant impact on the crystal properties.

Concerning the registered phase change between the crystalline and amorphous phase, one can notice that the Δφ are at least 50% smaller for the CAL-B-catalyzed copolyesters than for the materials synthesized using a heterogenous catalyst (97 vs. 188 deg in PBS-DLS 90-10; 111 vs. 156 deg in PBS-DLS 70-30 and 46 vs. 145 deg in PBS-DLS 50-50). The increase in the content of DLS soft segments reduces the density of the center part of the spherulites in relation to the surrounding molten phase.

**Figure 12:**
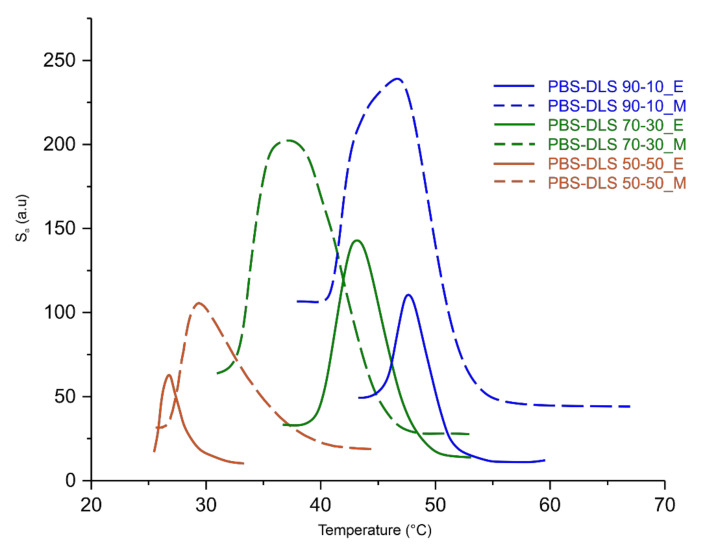
Roughness of the samples (S_a_) during cooling from the melt.

A change in the heterogeneity of the density of PBS-DLS copolyesters during spontaneous cooling with an exponential temperature decrease was observed. The roughness surface parameter S_a_ (expressed in degrees, see Figure 12) was in this case the difference in the optical way of each observed point in comparison to the arithmetical mean of the optical way for the entire surface. The maximum of the S_a_ parameter for CAL-B-catalyzed copolyesters was reached when about half of the test area was filled with crystalized material and the other half was still in the molten state. In the case of PBS-DLS copolyesters synthesized using a titanium dioxide/silicone dioxide catalyst, the crystallization process was not homogenous throughout the whole volume. Spherulites crystalized at different depths in the liquid, and in the phase image they overlap, causing the increase in difference of the optical way. This influenced the S_a_ maximum, which was for all PBS-DLS_M samples at least 30% higher than for PBS-DLS_E samples. A sharper signal peak of the crystallization curve is observed for PBS-DLS_E copolyesters, which indicates greater homogeneity of the formed crystalline phase as well. The crystallization started and finished at a higher temperature in the case of PBS-DLS_E with 70-30 and 90-10 wt% ratio; the exception was PBS-DLS 50-50_E, where the crystallization started and finished at a lower temperature compared to PBS-DLS_M.

**Figure 13:**
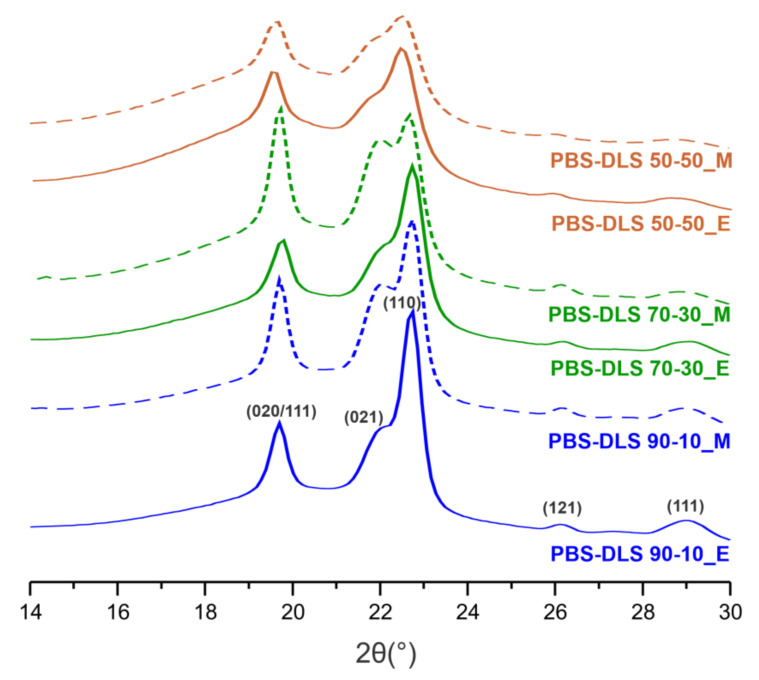
X-ray diffraction pattern of PBS-DLS copolymers.

To investigate the crystalline properties of the PBS-DLS copolyesters more deeply, X-ray diffraction (XRD) analysis was performed using melt-pressed thin polymer films. As presented in Figure 13, we noticed two strong reflections at 2θ = 19.7° (d = 4.50 Å,α (020/111)) and 22.8° (d = 3.90 Å,α (110)), shoulder at 2θ = 22.1° (d = 4.05 Å,α (021)), and two extra peaks appeared with lower intensities at 2θ = 26.3° (d = 3.38 Å,α (121)) and 28.9° (d = 3.09 Å,α (111)). Regarding the PBS-DLS copolyesters, they all demonstrate patterns typical for semicrystalline materials, and they can crystallize into a monoclinic α-form crystals [30,31]. Reflection peaks of the recorded crystalline fractions are located on the well-pronounced “bell-shaped” background, resulting from the presence of the amorphous phase. Considering the influence of soft segment content, one can notice that in the case of TiO_2_/SiO_2_-catalyzed PBS-DLS copolyester an intensity of peak at 2θ = 19.7° first, which slightly increases when we change the soft segment content from 10 to 30 wt%; then, it shifts to smaller angles when the soft segment content reaches 50 wt%. This phenomenon is not observed for CAL-B-catalyzed materials, where with the increasing amount of soft segments, the intensity of the peak at 2θ = 19.7° decreases gradually. Furthermore, the intensities of diffraction peaks located at 2θ = 22.8, 26.3 and 28.9° decrease with an increasing amount of soft segments, which is ascribed to the lower crystallinity of copolymers; this is also in a good agreement with previously described DSC results. From the XRD results mentioned above, the intensities of detected diffraction peaks change with the variable amount of amorphous DLS segments; however, they are still visible, which proves that the crystal structure of copolymers is preserved and corresponds to the typical lattice of α-PBS [31].

Moreover, the degree of crystallinity (X_c_’) was calculated from the XRD profiles by the ratio between the crystalline peaks area (A_c_) and diffraction area of all peaks (crystalline and amorphous) (A_t_) using Equation (12).

(12) $$X_{c}\prime =\frac{A_{c}}{A_{t}}\times 100\%$$

**Figure 14:**
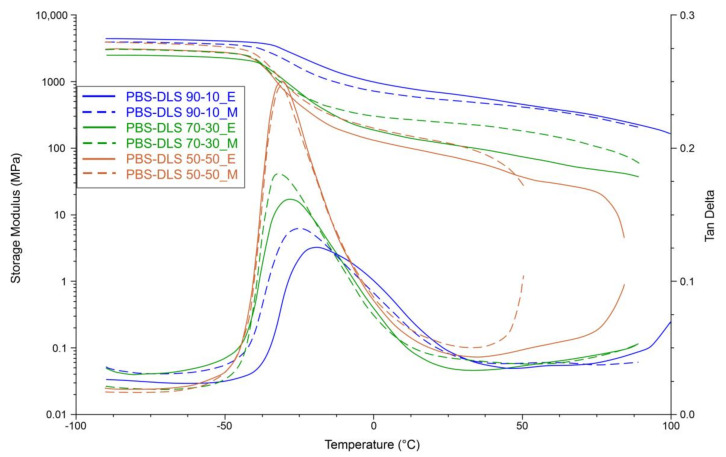
DMTA traces of the PBS-DLS copolyesters.

As evident from Figure 14, the relative area of amorphous halo increased with the increasing amount of DLS soft segments. Consequently, the degree of crystallinity (X_c_’) decreased, and the obtained results are in good agreement with the X_c_ values calculated from DSC data (see Table 3). Notably, PBS-DLS_E copolymers exhibit higher X_c_’, except for PBS-DLS 70-30_E, where slightly higher values were obtained for PBS-DLS 70-30_M (48.0 for PBS-DLS 70-30_E vs. 48.4 for PBS-DLS 70-30_M).

The effect of catalyst type and DLS soft segment content on the dynamic mechanical properties of PBS-DLS copolyesters was monitored in the tensile module at a starting temperature of −90 °C. Isochronal evolution of the storage modulus with temperature as well as temperature dependence of tan δ for PBS-DLS copolyesters is presented on Figure 14.

Recorded DMTA data indicate the viscoelastic properties of copolyesters. As can be seen, the storage modulus remains constant at temperatures below glass transition temperatures (−42 < T < −52 °C). Subsequently, a decrease in storage modulus (*E’)* values was noticed when the δ transition (T_δ_) in the amorphous phase occurred. T_δ_ relates to material composition; it is in the range between −37 and −27 °C and, by the correlation with the DSC results (Table 3), can be reasonably ascribed to T_g_. Upon further temperature increase, storage modulus decreased gradually until the melting temperature (T_m_) was reached. Obtained results show that both *E’* and peak magnitude of tan δ (T_δ_) tended to decrease with increasing soft segment content; this effect was expected, as we introduced a greater amount of amorphous phase in which molecular motion was not restricted by hard crystalline domains. Additionally, δ relaxation demonstrated by the tan δ curve became sharper, and its intensity increased when we changed the soft segment content from 10 to 30 or 50 wt%, which indicates an increased elastic response. Furthermore, by comparing PBS-DLS copolyesters synthesized using a CAL-B and TiO_2_/SiO_2_ catalyst, we can observe that the *E’* values of PBS-DLS_M with 70-30 and 50-50 wt% ratio are greater than that of PBS-DLS_E. The exception is PBS-DLS 90-10 copolyesters, where a greater storage modulus was recorded for PBS-DLS 90-10_E. On the other hand, PBS-DLS_E copolyester exhibited greater T_δ_ values for all tested compositions.

## 4. Conclusions

In the present study, we evidence that enzymatic synthesis is a valuable approach for the synthesis of biobased poly(butylene succinate)-co-(dilinoleic succinate) (PBS-DLS) copolyesters. PBS-DLS with a hard to soft segment ratio from 90-10 to 50-50 wt% was synthesized via two-step polycondensation in diphenyl ether using *Candida antarctica* lipase B as a biocatalyst and was compared to materials synthesized via polycondensation in melt performed with a TiO_2_/SiO_2_ catalyst. ^1^H NMR analysis revealed that PBS-DLS molar ratios were very close to those estimated theoretically; however, a better match was obtained for enzymatically catalyzed copolyesters, which shows that this process can be controlled more precisely. Furthermore, based on the ^13^C NMR spectra average sequence length of hard and soft segments (L_H_ and L_S_, respectively), the degree of randomness (R) was estimated. All PBS-DLS_E copolyesters present a more blocky microstructure compared to PBS-DLS_M materials, since the calculated R value was less than 1. In addition, the average sequence length of hard segments (L_H_) was two times greater in comparison to PBS-DLS_M materials. GPC measurements revealed the number average molar masses, varying from 16,300 to 25,200 g/mol for PBS-DLS_E copolymers and from 19,700 to 54,500 g/mol for PBS-DLS_M as well as weight average molar masses ranging from 49,900 to 205,600 g/mol for PBS-DLS_E and from 57,900 to 171,300 g/mol for PBS-DLS_M. Additionally, the registered polydispersity index (Đ) reached values between 3.1 and 8.2 for PBS-DLS_E copolymers and between 2.3 and 3.3 for PBS-DLS_M materials; this discrepancy may be due to the fact that CAL-B exhibited a higher affinity to form hard segments.

Moreover, DSC analysis revealed one glass transition temperature (T_g_) for all copolymers, shifting to lower values while increasing the soft DLS segments content. The same trend was observed in the case of crystallization and melting temperatures (T_c_ and T_m_, respectively), since we are introducing a greater amount of amorphous phase. T_g_, T_c_ and T_m_ values were comparable for both copolymer series; however, the total crystalline phase content was greater in the case of enzymatically catalyzed materials, which is strongly related to the microstructure of PBS-DLS_E and its block structure, where the length of the hard domains responsible for crystalline properties is greater.

This feature was also clearly reflected in digital holographic microscope (DHM) measurements, where PBS-DLS_E spherulites with lamellar crystals radiating outwards from the center were observed and demonstrated alternative bright and dark bands (banded spherulites), which become less pronounced with increasing soft segment content. Notably, those structures were not visible in the case of PBS-DLS_M spherulites. Digital holographic microscopy also offers a possibility to visualize the entire crystallization process in real time. This enabled us to observe the heterogeneous crystallization of PBD-DLS_M samples, contrary to the homogeneous crystallization of PBS-DLS_E samples, which could be characterized with the roughness parameter S_a_.

XRD suggested a semicrystalline structure of materials, since they can crystallize into a monoclinic α-form crystals. Moreover, the total crystalline phase content values (X_c_’) calculated from XRD are comparable to those estimated from DSC (X_c_). The intensities of detected diffraction peaks change depending on the soft DLS segment content; however, the crystal structure corresponding to the typical lattice of α-PBS is still preserved. Considering DMTA measurements, all copolymers are semicrystalline materials with viscoelastic properties. Furthermore, both *E’* and peak magnitude of tan δ (T_δ_) tended to decrease with increasing soft segment content.

PBS-DLS copolymers could be a promising alternative for well-known materials such as PLA, PCL and PGA, which are widely used, especially in the biomedicine field. Besides interesting physiochemical properties, PBS-DLS can be synthesized using succinic acid (or its ester), butanediol and dilinoleic diol driven from renewable resources, which makes it a fully biobased material. Additional value is given by the possibility of producing these materials using enzymes as a catalytic system, which strongly contributes to sustainable development. In this paper, we proved that the use of enzymes characterized by high regio-, enantio-, stereo-, and chemioselectivity is a valuable tool for designing materials with a specific microstructure and degree of crystallinity. This can be beneficial, especially in the case of biomedical, cosmetic, and pharmaceutical fields, where optically pure materials with well-defined structure are needed.

## Figures and Tables

**Table 1:** Average sequence length of hard and soft segments and degree of randomness.

Copolymer	R ^a^	L_H_ ^b^	L_S_ ^b^
PBS-DLS 90-10_E	0.82	9.33	1.40
PBS-DLS 90-10_M	0.98	7.22	1.19
PBS-DLS 70-30_E ^c^	0.66	6.88	1.93
PBS-DLS 70-30_M ^c^	0.87	3.01	1.85
PBS-DLS 50-50_E	0.47	5.07	3.66
PBS-DLS 50-50_M	0.83	2.04	2.96

^a^—degree of randomness calculated from Equation (3); ^b^—average sequence length calculated from Equations (1) and (2); ^c^—numerical values taken from Sokołowska et al. [18].

**Table 2:** Composition of PBS-DLS copolyesters determined from ^1^H NMR and GPC.

Copolymer	Composition		^1^H NMR ^a^	GPC ^b^		
	Theoretical wt% [mol%]	Calculated ^a^ wt% [mol%]	M_n_ [g/mol]	M_n_ [g/mol]	M_w_ [g/mol]	Đ
PBS-DLS 90-10_E	90/10 [97.0/3.0]	88/12 [96.5/3.5]	15,730	19,600	101,000	5.2
PBS-DLS 90-10_M		87/13 [96.0/4.0]	42,130	54,500	122,700	2.3
PBS-DLS 70-30_E^c^	70/30 [89.4/10.6]	68/32 [88.6/11.4]	33,610 ^c^	25,200	205,600	8.2
PBS-DLS 70-30_M^c^		64/36 [86.7/13.3]	53,530 ^c^	51,400	171,300	3.3
PBS-DLS 50-50_E	50/50 [78.5/21.5]	47/53 [76.2/23.8]	18,660	16,300	49,900	3.1
PBS-DLS 50-50_M		46/54 [75.6/24.4]	52,290	19,700	57,900	2.9

M_n_—number average molecular mass, M_w_—weight average molecular mass, Đ—dispersity index, ^a^ values calculated from ^1^H NMR ^b^ values determined from GPC. ^c^numerical values taken from Sokołowska et al. [18].

**Table 3:** DSC results for PBS-DLS copolymer series.

Copolymer	T_g_ (°C)	T_δ_ (°C)	T_c_ (°C)	ΔH_c_ (J/g)	T_cc_ (°C)	ΔH_cc_ (J/g)	T_m_ (°C)	ΔH_m_ (J/g)	X_c_ (%)	X_c_’ (%)
PBS-DLS 90-10_E	−42	−27	68	68.12	-	-	107	61.42	55.7	51.9
PBS-DLS 90-10_M	−44	−33	51	59.91	-	-	107	61.02	55.3	51.1
PBS-DLS 70-30_E ^a^	−45	−35	53	50.99	-	-	98	49.52	44.9	48.0
PBS-DLS 70-30_M ^a^	−47	−37	33	46.09	-	-	99	44.29	40.2	48.4
PBS-DLS 50-50_E	−49	−36	29	37.97	−6	1.61	83	40.59	36.8	38.0
PBS-DLS 50-50_M	−52	−36	14	24.03	0	11.66	82	38.91	35.3	36.6

T_g_—glass transition temperature; T_δ_—glass transition temperature from DMTA (determined as max. of tan δ); ΔC_p_—heat capacity at T_g_; ΔH_m_—melting enthalpy of the hard segments; T_m_—melting temperature; T_c_—crystallization temperature; X_c_—total crystalline phase content in the polymer calculated from DSC; X_c_’—total crystalline phase content in the polymer calculated from XRD; ^a^ numerical values taken from [18].

## Data Availability

The data presented in this study are available on request from the corresponding author.
